# Oleanolic acid ethanol monosolvate

**DOI:** 10.1107/S1600536810039929

**Published:** 2010-10-09

**Authors:** Anna Froelich, Andrzej K. Gzella

**Affiliations:** a Department of Organic Chemistry, Poznan University of Medical Sciences, ul. Grunwaldzka 6, 60-780 Poznań, Poland; b Faculty of Pharmacy, Ludwik Rydygier Collegium Medicum in Bydgoszcz, Nicolaus Copernicus University in Torun, ul. M. Curie Skłodowskiej 9, 85-094 Bydgoszcz, Poland

## Abstract

Crystals of the title compound (systematic name: 3β-hy­droxy­olean-12-en-28-oic acid ethanol monosolvate), C_30_H_48_O_3_·C_2_H_5_OH, were obtained from unsuccessful co-crystallization trials. The asymmetric unit contains two symmetry-independent oleanolic acid mol­ecules, as well as two ethanol solvent mol­ecules. Inter­molecular O—H⋯O hydrogen bonds stabilize the crystal packing. In the oleanolic acid mol­ecules, ring *C* has a slightly distorted envelope conformation, while rings *A*, *B*, *D* and *E* adopt chair conformations and rings *D* and *E* are *cis*-fused. Both independent ethanol mol­ecules are orientationally disordered [occupancy ratios of 0.742 (8):0.258 (8) and 0.632 (12):0.368 (12).

## Related literature

For the biological activity of oleanolic acid and its derivatives, see: Liu (1995 ▶, 2005 ▶). For 1a,3a-dimethylcyclohexane, see: Spirlet *et al.* (1980 ▶). For a description of the Cambridge Structural Database, see: Allen (2002 ▶). For ring conformation analysis, see: Cremer & Pople (1975 ▶).
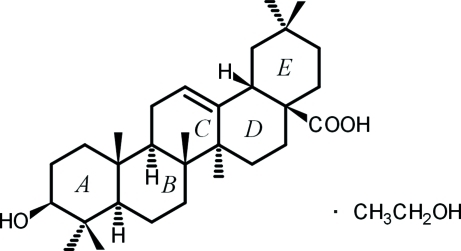


## Experimental

### 

#### Crystal data

C_30_H_48_O_3_·C_2_H_6_O
*M* *_r_* = 502.75
Monoclinic, 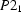

*a* = 16.3616 (14) Å
*b* = 7.2587 (5) Å
*c* = 25.786 (2) Å
β = 107.500 (9)°
*V* = 2920.7 (4) Å^3^
*Z* = 4
Cu *K*α radiation
μ = 0.57 mm^−1^
*T* = 130 K
0.34 × 0.11 × 0.08 mm

#### Data collection

Oxford Diffraction SuperNova Atlas diffractometer
Absorption correction: multi-scan (*CrysAlis PRO*; Oxford Diffraction, 2007 ▶) *T*_min_ = 0.901, *T*_max_ = 1.000
21878 measured reflections
11079 independent reflections
10744 reflections with *I* > 2σ(*I*)
*R*_int_ = 0.025

#### Refinement

*R*[*F* ^2^ > 2σ(*F* ^2^)] = 0.037
*wR*(*F* ^2^) = 0.114
*S* = 1.09
11079 reflections
732 parameters
1 restraint
H atoms treated by a mixture of independent and constrained refinement
Δρ_max_ = 0.22 e Å^−3^
Δρ_min_ = −0.20 e Å^−3^
Absolute structure: Flack (1983 ▶), 4907 Friedel pairs
Flack parameter: 0.01 (13)

### 

Data collection: *CrysAlis PRO* (Oxford Diffraction, 2007 ▶); cell refinement: *CrysAlis PRO*; data reduction: *CrysAlis PRO*; program(s) used to solve structure: *SHELXS97* (Sheldrick, 2008 ▶); program(s) used to refine structure: *SHELXL97* (Sheldrick, 2008 ▶); molecular graphics: *ORTEP-3 for Windows* (Farrugia, 1997 ▶); software used to prepare material for publication: *WinGX* (Farrugia, 1999 ▶) and *PLATON* (Spek, 2009 ▶).

## Supplementary Material

Crystal structure: contains datablocks I, global. DOI: 10.1107/S1600536810039929/bt5364sup1.cif

Structure factors: contains datablocks I. DOI: 10.1107/S1600536810039929/bt5364Isup2.hkl

Additional supplementary materials:  crystallographic information; 3D view; checkCIF report

## Figures and Tables

**Table 1:** Hydrogen-bond geometry (Å, °)

*D*—H⋯*A*	*D*—H	H⋯*A*	*D*⋯*A*	*D*—H⋯*A*
O1*BA*—H1*BO*⋯O1*B*	0.84 (3)	1.81 (3)	2.652 (2)	177 (3)
O1*AA*—H1*AO*⋯O1*A*	0.82	1.98	2.794 (7)	170
O1*A*—H11⋯O2*B*	0.85 (3)	2.02 (3)	2.8503 (18)	168 (3)
O1*B*—H12⋯O2*A*^i^	0.83 (3)	1.89 (3)	2.7204 (18)	174 (3)
O3*A*—H31⋯O1*BA*^ii^	0.95 (3)	1.61 (3)	2.552 (2)	177 (3)
O3*B*—H32⋯O1*AA*	0.96 (4)	1.64 (4)	2.575 (7)	163 (4)
C15*A*—H15*A*⋯O3*A*	0.97	2.58	3.1222 (19)	116
C15*B*—H15*C*⋯O2*B*	0.97	2.60	3.152 (2)	116
C23*A*—H23*A*⋯O2*B*	0.96	2.54	3.375 (2)	145

Symmetry codes: (i) 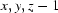; (ii) 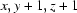.

**Fig. 1.**
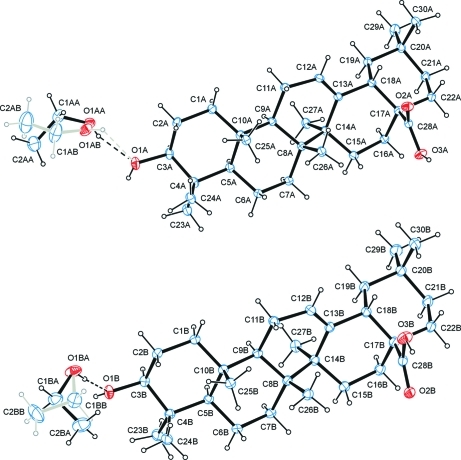
The independent molecules of oleanolic acid and ethanol showing the atomic labelling scheme. Non-H atoms are drawn as 30% probability displacement ellipsoids and H atoms are drawn as spheres of an arbitrary size.

**Fig. 2.**
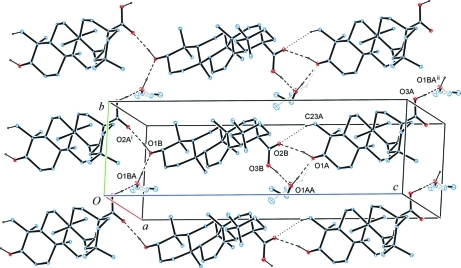
The hydrogen bonding (dashed and dotted lines) in the title structure. Symmetry codes: (i) x, y, -1 + z; (ii) x, 1 + y, 1 + z. The H atoms not involved in hydrogen bonds have been omitted for clarity.

## supplementary crystallographic information

### Comment

Oleanolic acid is a pentacyclic triterpenoid occurring abundantly in plants as a glycoside or a free compound. Oleanolic acid and some of its derivatives, both natural and semi-synthetic, show interesting biological activity which is the reason for the extensive studies on this class of compounds (Liu, 1995; 2005). There are 135 structures deposited in CSD Database, version 5.31 (Allen, 2002). However, the X-ray analysis of the structure of oleanolic acid has not been undertaken because of the difficulties with obtaining a crystal suitable for the investigations.

The structure presented in this paper was solved for the crystal obtained during the unsuccessful co-crystallization attempts. Crystallization process was carried out using ethanol with the addition of 12*α*-bromo-3a-aza-*A*-homo-18*β*-olean-28,13*β*-olide. The impact of the latter one on the crystals size requires further investigations.

Oleanolic acid crystallizes in form of an ethanol solvate. The asymmetric unit contains two symmetry-independent triterpenoid molecules (host) and two ethanol molecules (guest). Both solvent molecules are orientationally disordered. The figure 1 shows that in ethanol molecule A all atoms whereas in molecule B only methylene and methyl groups are split into two alternative positions. In molecule A the non-H atoms assigned to O1AA, C1AA and C2AA positions have the site occupancy factor of 63% whereas in molecule B the carbon atoms assigned to C1BA and C2BA positions have the factor of 74%.

The conformational differences between the symmetry-independent oleanolic acid molecules A and B are significant only in the angular arrangement of the carboxylic group. In molecule A, the carbonyl group C28═O2 adopts conformation halfway between synperiplanar and synclinal (-*sp*/-*sc*) with respect to C17—C18 bond [torsional angle C18A—C17A—C28A—O2A: -27.6 (2)°]. In molecule B, the conformation is halfway between anticlinal and antiperiplanar (+*ac*/+*ap*)[torsional angle: C18B—C17B—C28B—O2B: 145.84 (16)°]. The angular orientation of the carboxylic group in both independent molecules A and B is stabilized by the intermolecular hydrogen bonds in which this group acts both as a proton donor and acceptor (Fig. 2, Table 1).

In both independent molecules, rings *A*, *B*, *D* and *E* have chair conformation distorted to a different degree. Ring *C* assumes an envelope conformation [Cremer & Pople (1975) parameters molecule A: *Q* = 0.565 (2) Å, θ = 51.1 (2)°, φ = 1.7 (2)°; molecule B: *Q* = 0.544 (2) Å, θ = 50.5 (2)°, φ = 4.5 (3)°].

The dihedral angles *A*/*B*, *B*/*C*, *C*/*D* and *D*/*E* in molecule A are 17.41 (8), 13.75 (10), 14.13 (10) and 57.92 (6)°, while in molecule B are 14.68 (10), 12.34 (11), 17.47 (10) and 60.26 (6)°. Rings *A*/*B* and *B*/*C* are *trans*-, whereas rings *D*/*E* are *cis*-fused. Each of the axial methyl groups C24, C25 and C26 repulses the adjacent methyls. This results in the lengthening of the interatomic distances C24···C25 and C25···C26 [molecule A: 3.2434 (23) and 3.2648 (23) Å; molecule B: 3.2645 (25) and 3.1958 (24) Å] in comparison with the undistorted molecule of 1a,3a-dimethylcyclohexane in which the distance between the methyl carbons is merely 2.52 Å [Spirlet *et al.*, 1980]. An additional consequence of the repulsive interactions between the methyl groups is deformation of the angles C4—C5—C10 and C8—C9—C10 to the values of 117.30 (13) and 117.33 (12)° (molecule A) and 117.90 (13) and 116.98 (13)° (molecule B).

The hydroxyl group at C3 in molecules A and B of oleanolic acid is equatorial with respect to ring *A* and adopts β orientation.

In the crystal lattice, the symmetry-independent molecules A and B of oleanolic acid are connected with hydrogen bonds O1A-H11···O2B and O1B-H12···O2A^i^ (Fig. 2, Table 1) into infinite chains extending along the *c* axis. The hydrogen bonds involve the C3 hydroxyl group and the carbonyl oxygen atom of the C17 carboxylic group. Both of the mentioned functional groups form also hydrogen bonds with the solvent molecules. The ethanol molecules marked A are hydrogen bonded (O1AA-H1AO···O1A and O3B-H32···O1AA) to the triterpenoid molecules A and B belonging to the same chain, whereas the ethanol molecules B are linked through the O3A—H31···O1BA^ii^ and O1BA-H1BO···O1B hydrogen bonds with the triterpenoide molecules A and B but from the adjacent chains (Fig. 2, Table 1). Therefore, the ethanol molecules B connect the chains of triterpenoid molecules into two-dimensional layers that extend parallel to the *bc* plane. The layer thickness is about half of the *a* parameter length.

### Experimental

Oleanolic acid was extracted from mistletoe leaves. Equimolar quantities of oleanolic acid and 12*α*-bromo-3a-aza-*A*-homo-18*β*-olean-28,13*β*-olide as an additive were dissolved together in hot ethanol and the mixture was set aside to crystallize at room temperature.

### Refinement

Except for the triterpenoide hydroxyl and carboxylic groups H atoms which were refined freely the remaining H atoms were positioned into the idealized positions and were refined within the riding model approximation: C_methyl_—H = 0.96 Å, C_methylene_—H = 0.97 Å, C_methine_—H = 0.98 Å, C(*sp*^2^)—H = 0.93 Å, O—H = 0.82 Å; *U*_iso_(H) = 1.2*U*_eq_(C) or 1.5*U*_eq_(C) for methyl H. The methyl atoms were refined as a rigid group, which was allowed to rotate. 4907 Friedel pairs were used to determine the absolute configuration which is also known by reference to the chirality of the oleanane derivatives.

### Crystal data

**Table:**

C_30_H_48_O_3_·C_2_H_6_O	*F*(000) = 1112
*M**_r_* = 502.75	*D*_x_ = 1.143 Mg m^−^^3^
Monoclinic, *P*2_1_	Melting point = 582–583 K
Hall symbol: P 2yb	Cu *K*α radiation, λ = 1.54184 Å
*a* = 16.3616 (14) Å	Cell parameters from 11811 reflections
*b* = 7.2587 (5) Å	θ = 2.8–75.2°
*c* = 25.786 (2) Å	µ = 0.56 mm^−^^1^
β = 107.500 (9)°	*T* = 130 K
*V* = 2920.7 (4) Å^3^	Prism, colourless
*Z* = 4	0.34 × 0.11 × 0.08 mm

### Data collection

**Table:**

Oxford Diffraction SuperNova Atlas diffractometer	11079 independent reflections
Radiation source: SuperNova (Cu) X-ray Source	10744 reflections with *I* > 2σ(*I*)
mirror	*R*_int_ = 0.025
Detector resolution: 10.5357 pixels mm^-1^	θ_max_ = 72.1°, θ_min_ = 8.9°
ω scans	*h* = −20→19
Absorption correction: multi-scan (*CrysAlis PRO*; Oxford Diffraction, 2007)	*k* = −8→8
*T*_min_ = 0.901, *T*_max_ = 1.000	*l* = −23→31
21878 measured reflections	

### Refinement

**Table:**

Refinement on *F*^2^	Secondary atom site location: difference Fourier map
Least-squares matrix: full	Hydrogen site location: inferred from neighbouring sites
*R*[*F*^2^ > 2σ(*F*^2^)] = 0.037	H atoms treated by a mixture of independent and constrained refinement
*wR*(*F*^2^) = 0.114	*w* = 1/[σ^2^(*F*_o_^2^) + (0.0678*P*)^2^ + 0.6434*P*] where *P* = (*F*_o_^2^ + 2*F*_c_^2^)/3
*S* = 1.09	(Δ/σ)_max_ = 0.001
11079 reflections	Δρ_max_ = 0.22 e Å^−^^3^
732 parameters	Δρ_min_ = −0.20 e Å^−^^3^
1 restraint	Absolute structure: Flack (1983), 4907 Friedel pairs
Primary atom site location: structure-invariant direct methods	Flack parameter: 0.01 (13)

### Special details

**Table:**

Geometry. All e.s.d.'s (except the e.s.d. in the dihedral angle between two l.s. planes) are estimated using the full covariance matrix. The cell e.s.d.'s are taken into account individually in the estimation of e.s.d.'s in distances, angles and torsion angles; correlations between e.s.d.'s in cell parameters are only used when they are defined by crystal symmetry. An approximate (isotropic) treatment of cell e.s.d.'s is used for estimating e.s.d.'s involving l.s. planes.
Refinement. Refinement of *F*^2^ against ALL reflections. The weighted *R*-factor *wR* and goodness of fit *S* are based on *F*^2^, conventional *R*-factors *R* are based on *F*, with *F* set to zero for negative *F*^2^. The threshold expression of *F*^2^ > σ(*F*^2^) is used only for calculating *R*-factors(gt) *etc*. and is not relevant to the choice of reflections for refinement. *R*-factors based on *F*^2^ are statistically about twice as large as those based on *F*, and *R*- factors based on ALL data will be even larger.

### Fractional atomic coordinates and isotropic or equivalent isotropic displacement parameters (Å^2^)

**Table:**

	*x*	*y*	*z*	*U*_iso_*/*U*_eq_	Occ. (<1)
O1A	0.90339 (8)	0.6221 (2)	0.57647 (5)	0.0313 (3)	
O2A	0.77763 (8)	0.93170 (18)	0.96632 (5)	0.0301 (3)	
O3A	0.70930 (8)	1.18974 (18)	0.93395 (5)	0.0316 (3)	
C1A	0.86540 (10)	0.5160 (2)	0.70946 (6)	0.0238 (3)	
H1A	0.8969	0.4346	0.7384	0.029*	
H1B	0.8074	0.4688	0.6956	0.029*	
C2A	0.90733 (10)	0.5118 (2)	0.66370 (7)	0.0256 (3)	
H2A	0.9673	0.5452	0.6782	0.031*	
H2B	0.9042	0.3879	0.6491	0.031*	
C3A	0.86311 (10)	0.6437 (3)	0.61866 (6)	0.0249 (3)	
H3A	0.8032	0.6049	0.6039	0.030*	
C4A	0.86382 (10)	0.8445 (2)	0.63751 (6)	0.0229 (3)	
C5A	0.82363 (9)	0.8450 (2)	0.68520 (6)	0.0208 (3)	
H5A	0.7647	0.8019	0.6688	0.025*	
C6A	0.81396 (10)	1.0361 (2)	0.70722 (6)	0.0232 (3)	
H6A	0.7964	1.1231	0.6774	0.028*	
H6B	0.8686	1.0764	0.7315	0.028*	
C7A	0.74730 (10)	1.0319 (2)	0.73782 (6)	0.0231 (3)	
H7A	0.6918	1.0038	0.7123	0.028*	
H7B	0.7438	1.1534	0.7526	0.028*	
C8A	0.76693 (10)	0.8902 (2)	0.78446 (6)	0.0201 (3)	
C9A	0.79544 (10)	0.7030 (2)	0.76614 (6)	0.0202 (3)	
H9A	0.7433	0.6517	0.7407	0.024*	
C10A	0.86222 (10)	0.7093 (2)	0.73320 (6)	0.0202 (3)	
C11A	0.81932 (11)	0.5671 (2)	0.81433 (7)	0.0261 (3)	
H11A	0.8131	0.4423	0.8002	0.031*	
H11B	0.8791	0.5850	0.8348	0.031*	
C12A	0.76539 (10)	0.5886 (2)	0.85225 (6)	0.0231 (3)	
H12A	0.7752	0.5053	0.8809	0.028*	
C13A	0.70544 (10)	0.7145 (2)	0.84866 (6)	0.0202 (3)	
C14A	0.68265 (10)	0.8523 (2)	0.80123 (6)	0.0210 (3)	
C15A	0.64668 (11)	1.0357 (2)	0.81549 (6)	0.0236 (3)	
H15A	0.6943	1.1151	0.8338	0.028*	
H15B	0.6148	1.0963	0.7820	0.028*	
C16A	0.58848 (10)	1.0150 (2)	0.85147 (6)	0.0229 (3)	
H16A	0.5370	0.9491	0.8316	0.027*	
H16B	0.5716	1.1361	0.8604	0.027*	
C17A	0.63323 (10)	0.9107 (2)	0.90410 (6)	0.0216 (3)	
C18A	0.65581 (10)	0.7139 (2)	0.89009 (6)	0.0202 (3)	
H18A	0.6937	0.6606	0.9236	0.024*	
C19A	0.57587 (10)	0.5889 (2)	0.87175 (6)	0.0231 (3)	
H19A	0.5396	0.6318	0.8368	0.028*	
H19B	0.5941	0.4646	0.8668	0.028*	
C20A	0.52282 (10)	0.5835 (2)	0.91188 (6)	0.0242 (3)	
C21A	0.49777 (11)	0.7811 (2)	0.92099 (7)	0.0252 (3)	
H21A	0.4611	0.8302	0.8869	0.030*	
H21B	0.4652	0.7802	0.9468	0.030*	
C22A	0.57532 (11)	0.9061 (2)	0.94220 (7)	0.0247 (3)	
H22A	0.6090	0.8638	0.9779	0.030*	
H22B	0.5559	1.0301	0.9461	0.030*	
C23A	0.80474 (11)	0.9573 (3)	0.59050 (6)	0.0292 (4)	
H23A	0.8218	0.9398	0.5583	0.044*	
H23B	0.8090	1.0855	0.6001	0.044*	
H23C	0.7466	0.9169	0.5838	0.044*	
C24A	0.95432 (11)	0.9281 (3)	0.65146 (7)	0.0281 (4)	
H24A	0.9948	0.8453	0.6748	0.042*	
H24B	0.9554	1.0438	0.6697	0.042*	
H24C	0.9693	0.9473	0.6186	0.042*	
C25A	0.95356 (10)	0.7606 (2)	0.76792 (6)	0.0241 (3)	
H25A	0.9662	0.7012	0.8027	0.036*	
H25B	0.9576	0.8917	0.7729	0.036*	
H25C	0.9939	0.7209	0.7498	0.036*	
C26A	0.83793 (10)	0.9721 (2)	0.83296 (6)	0.0243 (3)	
H26A	0.8864	1.0052	0.8213	0.037*	
H26B	0.8550	0.8824	0.8615	0.037*	
H26C	0.8164	1.0798	0.8462	0.037*	
C27A	0.60956 (10)	0.7624 (3)	0.75467 (6)	0.0243 (3)	
H27A	0.6228	0.6351	0.7511	0.036*	
H27B	0.6044	0.8255	0.7211	0.036*	
H27C	0.5564	0.7714	0.7631	0.036*	
C28A	0.71465 (10)	1.0086 (2)	0.93722 (6)	0.0226 (3)	
C29A	0.44085 (12)	0.4713 (3)	0.88732 (8)	0.0340 (4)	
H29A	0.4083	0.4665	0.9127	0.051*	
H29B	0.4557	0.3486	0.8796	0.051*	
H29C	0.4072	0.5287	0.8543	0.051*	
C30A	0.57331 (12)	0.4954 (3)	0.96598 (7)	0.0299 (4)	
H30A	0.6275	0.5566	0.9800	0.045*	
H30B	0.5827	0.3674	0.9603	0.045*	
H30C	0.5414	0.5070	0.9915	0.045*	
O1B	0.81330 (9)	0.66050 (18)	0.04267 (5)	0.0304 (3)	
O2B	0.79977 (8)	0.7474 (2)	0.47278 (5)	0.0320 (3)	
O3B	0.71731 (10)	0.4977 (2)	0.45176 (6)	0.0428 (3)	
C1B	0.71248 (10)	0.5072 (2)	0.14822 (6)	0.0238 (3)	
H1C	0.6986	0.3838	0.1573	0.029*	
H1D	0.6594	0.5760	0.1356	0.029*	
C2B	0.75329 (11)	0.4953 (2)	0.10215 (6)	0.0254 (3)	
H2C	0.8040	0.4182	0.1135	0.031*	
H2D	0.7132	0.4385	0.0706	0.031*	
C3B	0.77771 (11)	0.6836 (2)	0.08688 (6)	0.0250 (3)	
H3B	0.7252	0.7568	0.0736	0.030*	
C4B	0.84041 (11)	0.7895 (2)	0.13473 (6)	0.0243 (3)	
C5B	0.80132 (10)	0.7879 (2)	0.18307 (6)	0.0219 (3)	
H5B	0.7488	0.8612	0.1701	0.026*	
C6B	0.85528 (11)	0.8903 (3)	0.23372 (7)	0.0264 (3)	
H6C	0.8776	1.0029	0.2229	0.032*	
H6D	0.9035	0.8141	0.2531	0.032*	
C7B	0.80119 (11)	0.9368 (2)	0.27121 (7)	0.0278 (4)	
H7C	0.7575	1.0250	0.2529	0.033*	
H7D	0.8377	0.9957	0.3037	0.033*	
C8B	0.75715 (10)	0.7698 (2)	0.28832 (6)	0.0237 (3)	
C9B	0.71504 (10)	0.6467 (2)	0.23786 (6)	0.0222 (3)	
H9B	0.6670	0.7197	0.2153	0.027*	
C10B	0.77086 (9)	0.5999 (2)	0.19971 (6)	0.0214 (3)	
C11B	0.67340 (11)	0.4763 (3)	0.25505 (7)	0.0281 (3)	
H11C	0.6292	0.4301	0.2236	0.034*	
H11D	0.7165	0.3808	0.2667	0.034*	
C12B	0.63430 (11)	0.5113 (3)	0.29992 (7)	0.0270 (3)	
H12B	0.6045	0.4147	0.3095	0.032*	
C13B	0.63866 (10)	0.6688 (3)	0.32707 (6)	0.0248 (3)	
C14B	0.68334 (10)	0.8376 (2)	0.31246 (6)	0.0247 (3)	
C15B	0.72356 (11)	0.9623 (3)	0.36257 (7)	0.0281 (3)	
H15C	0.7806	0.9167	0.3811	0.034*	
H15D	0.7297	1.0856	0.3497	0.034*	
C16B	0.67345 (12)	0.9750 (3)	0.40403 (7)	0.0302 (4)	
H16C	0.7077	1.0411	0.4359	0.036*	
H16D	0.6214	1.0450	0.3883	0.036*	
C17B	0.65013 (11)	0.7866 (3)	0.42139 (7)	0.0276 (4)	
C18B	0.59397 (10)	0.6814 (3)	0.37119 (6)	0.0269 (3)	
H18B	0.5878	0.5553	0.3830	0.032*	
C19B	0.50291 (11)	0.7641 (3)	0.35052 (7)	0.0298 (4)	
H19C	0.5068	0.8867	0.3365	0.036*	
H19D	0.4689	0.6894	0.3206	0.036*	
C20B	0.45584 (11)	0.7770 (3)	0.39395 (7)	0.0339 (4)	
C21B	0.51280 (12)	0.8853 (3)	0.44276 (7)	0.0373 (4)	
H21C	0.5174	1.0117	0.4318	0.045*	
H21D	0.4856	0.8869	0.4714	0.045*	
C22B	0.60269 (12)	0.8040 (3)	0.46524 (7)	0.0355 (4)	
H22C	0.5985	0.6830	0.4802	0.043*	
H22D	0.6362	0.8815	0.4946	0.043*	
C23B	0.84380 (13)	0.9892 (3)	0.11599 (7)	0.0320 (4)	
H23D	0.7892	1.0468	0.1110	0.048*	
H23E	0.8572	0.9903	0.0822	0.048*	
H23F	0.8871	1.0554	0.1430	0.048*	
C24B	0.93099 (11)	0.7092 (3)	0.14856 (7)	0.0316 (4)	
H24D	0.9544	0.7336	0.1193	0.047*	
H24E	0.9287	0.5786	0.1538	0.047*	
H24F	0.9666	0.7652	0.1813	0.047*	
C25B	0.84530 (10)	0.4654 (2)	0.22509 (7)	0.0260 (3)	
H25D	0.8289	0.3822	0.2491	0.039*	
H25E	0.8950	0.5338	0.2451	0.039*	
H25F	0.8583	0.3966	0.1968	0.039*	
C26B	0.82541 (11)	0.6599 (3)	0.33183 (7)	0.0289 (4)	
H26D	0.7996	0.5517	0.3416	0.043*	
H26E	0.8482	0.7354	0.3635	0.043*	
H26F	0.8709	0.6244	0.3175	0.043*	
C27B	0.61333 (11)	0.9524 (3)	0.27131 (7)	0.0287 (4)	
H27D	0.5782	1.0128	0.2900	0.043*	
H27E	0.5785	0.8724	0.2438	0.043*	
H27F	0.6400	1.0430	0.2546	0.043*	
C28B	0.73084 (11)	0.6762 (3)	0.45021 (6)	0.0285 (4)	
C29B	0.37235 (12)	0.8830 (4)	0.37015 (8)	0.0411 (5)	
H29D	0.3414	0.8879	0.3964	0.062*	
H29E	0.3381	0.8218	0.3379	0.062*	
H29F	0.3850	1.0059	0.3612	0.062*	
C30B	0.43526 (13)	0.5863 (4)	0.41156 (8)	0.0397 (4)	
H30D	0.4061	0.5987	0.4386	0.060*	
H30E	0.4875	0.5187	0.4265	0.060*	
H30F	0.3992	0.5215	0.3807	0.060*	
O1BA	0.84055 (9)	0.3450 (2)	−0.00132 (7)	0.0463 (4)	
H1BO	0.8341 (18)	0.446 (5)	0.0130 (12)	0.057 (8)*	
C1BA	0.9207 (2)	0.3265 (6)	−0.0114 (2)	0.0467 (10)	0.742 (8)
H1BA	0.9281	0.4274	−0.0342	0.056*	0.742 (8)
H1BB	0.9214	0.2124	−0.0308	0.056*	0.742 (8)
C2BA	0.9930 (2)	0.3264 (6)	0.04001 (16)	0.0608 (12)	0.742 (8)
H2BA	1.0461	0.3107	0.0318	0.091*	0.742 (8)
H2BB	0.9857	0.2270	0.0628	0.091*	0.742 (8)
H2BC	0.9940	0.4413	0.0586	0.091*	0.742 (8)
C1BB	0.9313 (7)	0.3021 (18)	0.0183 (6)	0.050 (3)	0.258 (8)
H1BC	0.9400	0.1799	0.0342	0.060*	0.258 (8)
H1BD	0.9611	0.3905	0.0457	0.060*	0.258 (8)
C2BB	0.9646 (8)	0.311 (2)	−0.0308 (5)	0.073 (4)	0.258 (8)
H2BD	1.0253	0.2893	−0.0194	0.109*	0.258 (8)
H2BE	0.9532	0.4314	−0.0471	0.109*	0.258 (8)
H2BF	0.9364	0.2197	−0.0568	0.109*	0.258 (8)
O1AA	0.8414 (4)	0.3086 (9)	0.5142 (3)	0.0323 (10)	0.632 (12)
H1AO	0.8655	0.3969	0.5323	0.048*	0.632 (12)
C1AA	0.9004 (3)	0.2115 (7)	0.49363 (15)	0.0420 (13)	0.632 (12)
H1AA	0.8820	0.0844	0.4868	0.050*	0.632 (12)
H1AB	0.9564	0.2121	0.5206	0.050*	0.632 (12)
C2AA	0.9069 (3)	0.2957 (11)	0.4424 (2)	0.0619 (17)	0.632 (12)
H2AA	0.9462	0.2258	0.4292	0.093*	0.632 (12)
H2AB	0.9271	0.4201	0.4494	0.093*	0.632 (12)
H2AC	0.8515	0.2957	0.4157	0.093*	0.632 (12)
O1AB	0.8662 (7)	0.3365 (18)	0.5130 (5)	0.039 (2)	0.368 (12)
H2AO	0.8859	0.3897	0.5422	0.058*	0.368 (12)
C1AB	0.9355 (7)	0.2945 (18)	0.4897 (4)	0.068 (3)	0.368 (12)
H1AC	0.9828	0.2370	0.5170	0.082*	0.368 (12)
H1AD	0.9562	0.4073	0.4779	0.082*	0.368 (12)
C2AB	0.9031 (6)	0.170 (3)	0.4434 (5)	0.084 (4)	0.368 (12)
H2AD	0.9482	0.1422	0.4279	0.125*	0.368 (12)
H2AE	0.8564	0.2272	0.4165	0.125*	0.368 (12)
H2AF	0.8835	0.0575	0.4555	0.125*	0.368 (12)
H11	0.8743 (19)	0.675 (5)	0.5475 (12)	0.061 (9)*	
H31	0.759 (2)	1.243 (5)	0.9581 (12)	0.065 (9)*	
H12	0.8009 (16)	0.748 (4)	0.0208 (10)	0.042 (6)*	
H32	0.770 (2)	0.444 (6)	0.4732 (14)	0.089 (11)*	

### Atomic displacement parameters (Å^2^)

**Table:**

	*U*^11^	*U*^22^	*U*^33^	*U*^12^	*U*^13^	*U*^23^
O1A	0.0295 (6)	0.0428 (8)	0.0240 (6)	0.0069 (6)	0.0118 (5)	−0.0030 (5)
O2A	0.0295 (6)	0.0258 (6)	0.0305 (6)	−0.0034 (5)	0.0021 (5)	0.0030 (5)
O3A	0.0313 (6)	0.0205 (6)	0.0394 (7)	−0.0025 (5)	0.0052 (5)	−0.0034 (5)
C1A	0.0218 (7)	0.0241 (8)	0.0277 (7)	−0.0007 (6)	0.0105 (6)	−0.0010 (6)
C2A	0.0239 (7)	0.0257 (8)	0.0292 (8)	0.0016 (6)	0.0111 (6)	−0.0051 (7)
C3A	0.0196 (7)	0.0328 (9)	0.0247 (8)	−0.0009 (6)	0.0101 (6)	−0.0053 (7)
C4A	0.0186 (7)	0.0296 (8)	0.0221 (7)	−0.0002 (6)	0.0085 (6)	−0.0002 (6)
C5A	0.0175 (7)	0.0239 (8)	0.0215 (7)	−0.0035 (6)	0.0067 (6)	−0.0013 (6)
C6A	0.0272 (8)	0.0208 (8)	0.0229 (7)	−0.0013 (6)	0.0095 (6)	0.0022 (6)
C7A	0.0273 (8)	0.0202 (7)	0.0233 (7)	0.0028 (6)	0.0100 (6)	0.0018 (6)
C8A	0.0204 (7)	0.0210 (7)	0.0191 (7)	0.0002 (6)	0.0061 (6)	0.0006 (6)
C9A	0.0199 (7)	0.0200 (7)	0.0218 (7)	−0.0009 (6)	0.0081 (6)	0.0011 (6)
C10A	0.0185 (7)	0.0198 (7)	0.0225 (7)	−0.0012 (6)	0.0066 (6)	−0.0020 (6)
C11A	0.0274 (8)	0.0240 (8)	0.0311 (8)	0.0048 (6)	0.0152 (6)	0.0055 (7)
C12A	0.0256 (8)	0.0214 (7)	0.0238 (7)	0.0018 (6)	0.0098 (6)	0.0055 (6)
C13A	0.0209 (7)	0.0196 (7)	0.0202 (7)	−0.0029 (6)	0.0063 (5)	−0.0007 (6)
C14A	0.0214 (7)	0.0216 (8)	0.0209 (7)	0.0007 (6)	0.0079 (6)	0.0007 (6)
C15A	0.0267 (8)	0.0230 (8)	0.0221 (7)	0.0033 (6)	0.0088 (6)	0.0044 (6)
C16A	0.0242 (7)	0.0207 (7)	0.0243 (7)	0.0044 (6)	0.0081 (6)	0.0011 (6)
C17A	0.0225 (7)	0.0221 (8)	0.0214 (7)	−0.0004 (6)	0.0082 (6)	−0.0003 (6)
C18A	0.0210 (7)	0.0202 (7)	0.0201 (7)	0.0012 (6)	0.0076 (5)	0.0021 (6)
C19A	0.0251 (8)	0.0229 (7)	0.0225 (7)	−0.0019 (6)	0.0091 (6)	−0.0023 (6)
C20A	0.0235 (8)	0.0266 (8)	0.0255 (7)	−0.0053 (7)	0.0117 (6)	−0.0037 (7)
C21A	0.0231 (8)	0.0281 (8)	0.0279 (8)	0.0003 (6)	0.0128 (6)	−0.0020 (6)
C22A	0.0286 (8)	0.0230 (8)	0.0256 (7)	−0.0006 (6)	0.0129 (6)	−0.0030 (6)
C23A	0.0297 (8)	0.0365 (9)	0.0224 (7)	0.0047 (7)	0.0094 (6)	0.0014 (7)
C24A	0.0237 (8)	0.0358 (10)	0.0273 (8)	−0.0051 (7)	0.0112 (6)	0.0003 (7)
C25A	0.0207 (7)	0.0282 (8)	0.0235 (7)	−0.0025 (6)	0.0070 (6)	−0.0006 (6)
C26A	0.0251 (8)	0.0256 (8)	0.0229 (7)	−0.0055 (6)	0.0081 (6)	−0.0028 (6)
C27A	0.0195 (7)	0.0320 (8)	0.0219 (7)	−0.0014 (6)	0.0072 (6)	−0.0001 (6)
C28A	0.0273 (8)	0.0229 (8)	0.0197 (7)	−0.0009 (7)	0.0104 (6)	−0.0003 (6)
C29A	0.0283 (8)	0.0382 (10)	0.0398 (9)	−0.0109 (8)	0.0167 (7)	−0.0104 (8)
C30A	0.0353 (9)	0.0302 (9)	0.0298 (8)	0.0012 (7)	0.0180 (7)	0.0045 (7)
O1B	0.0434 (7)	0.0258 (6)	0.0245 (6)	0.0068 (5)	0.0143 (5)	0.0036 (5)
O2B	0.0263 (6)	0.0409 (7)	0.0272 (6)	−0.0008 (5)	0.0057 (5)	−0.0005 (5)
O3B	0.0427 (8)	0.0333 (8)	0.0437 (8)	−0.0025 (6)	0.0001 (6)	0.0025 (6)
C1B	0.0209 (7)	0.0242 (8)	0.0246 (7)	−0.0006 (6)	0.0045 (6)	−0.0002 (6)
C2B	0.0255 (8)	0.0257 (9)	0.0229 (7)	0.0007 (7)	0.0039 (6)	−0.0040 (6)
C3B	0.0266 (8)	0.0249 (8)	0.0229 (7)	0.0048 (6)	0.0068 (6)	0.0016 (6)
C4B	0.0239 (8)	0.0239 (8)	0.0249 (7)	−0.0008 (6)	0.0071 (6)	0.0001 (6)
C5B	0.0176 (7)	0.0241 (8)	0.0225 (7)	0.0022 (6)	0.0037 (6)	0.0000 (6)
C6B	0.0222 (8)	0.0299 (9)	0.0264 (8)	−0.0058 (7)	0.0061 (6)	−0.0024 (7)
C7B	0.0282 (8)	0.0266 (9)	0.0284 (8)	−0.0073 (7)	0.0080 (7)	−0.0071 (7)
C8B	0.0236 (8)	0.0243 (8)	0.0226 (7)	−0.0005 (6)	0.0061 (6)	−0.0010 (6)
C9B	0.0196 (7)	0.0215 (8)	0.0237 (7)	−0.0004 (6)	0.0040 (6)	0.0004 (6)
C10B	0.0184 (7)	0.0214 (7)	0.0226 (7)	0.0025 (6)	0.0035 (6)	0.0000 (6)
C11B	0.0295 (8)	0.0260 (8)	0.0317 (8)	−0.0042 (7)	0.0135 (7)	−0.0038 (7)
C12B	0.0261 (8)	0.0285 (9)	0.0277 (8)	−0.0025 (7)	0.0100 (6)	0.0016 (7)
C13B	0.0225 (7)	0.0297 (8)	0.0207 (7)	0.0008 (6)	0.0043 (6)	0.0018 (6)
C14B	0.0245 (8)	0.0250 (8)	0.0245 (7)	0.0008 (7)	0.0073 (6)	−0.0009 (6)
C15B	0.0304 (8)	0.0267 (8)	0.0278 (8)	−0.0033 (7)	0.0097 (7)	−0.0035 (7)
C16B	0.0290 (8)	0.0339 (10)	0.0270 (8)	0.0023 (7)	0.0072 (6)	−0.0059 (7)
C17B	0.0239 (8)	0.0352 (9)	0.0230 (7)	0.0007 (7)	0.0060 (6)	−0.0027 (7)
C18B	0.0233 (8)	0.0348 (9)	0.0223 (7)	0.0004 (7)	0.0062 (6)	0.0005 (7)
C19B	0.0246 (8)	0.0402 (10)	0.0232 (7)	−0.0005 (7)	0.0052 (6)	−0.0023 (7)
C20B	0.0254 (8)	0.0498 (12)	0.0268 (8)	−0.0006 (8)	0.0082 (7)	−0.0050 (8)
C21B	0.0282 (9)	0.0572 (13)	0.0275 (8)	0.0007 (9)	0.0101 (7)	−0.0102 (8)
C22B	0.0284 (9)	0.0540 (13)	0.0234 (8)	−0.0024 (8)	0.0070 (7)	−0.0078 (8)
C23B	0.0405 (10)	0.0260 (9)	0.0310 (8)	−0.0054 (8)	0.0129 (7)	0.0006 (7)
C24B	0.0237 (8)	0.0418 (10)	0.0305 (8)	0.0013 (7)	0.0098 (6)	0.0011 (7)
C25B	0.0234 (8)	0.0275 (8)	0.0263 (7)	0.0031 (7)	0.0061 (6)	0.0023 (7)
C26B	0.0236 (8)	0.0362 (9)	0.0252 (8)	0.0037 (7)	0.0048 (6)	−0.0010 (7)
C27B	0.0303 (8)	0.0285 (8)	0.0282 (8)	0.0067 (7)	0.0101 (7)	0.0025 (7)
C28B	0.0284 (8)	0.0366 (10)	0.0205 (7)	0.0000 (7)	0.0076 (6)	−0.0013 (7)
C29B	0.0262 (9)	0.0610 (14)	0.0351 (9)	0.0027 (9)	0.0076 (7)	−0.0065 (9)
C30B	0.0321 (9)	0.0582 (13)	0.0307 (9)	−0.0052 (9)	0.0122 (7)	0.0011 (9)
O1BA	0.0316 (7)	0.0417 (9)	0.0636 (10)	−0.0043 (6)	0.0111 (7)	−0.0254 (8)
C1BA	0.0359 (18)	0.052 (2)	0.052 (2)	−0.0029 (14)	0.0139 (19)	−0.0133 (18)
C2BA	0.038 (2)	0.065 (2)	0.071 (2)	0.0032 (16)	0.0046 (18)	−0.0057 (19)
C1BB	0.035 (7)	0.067 (7)	0.049 (6)	0.016 (4)	0.012 (6)	−0.002 (6)
C2BB	0.079 (8)	0.079 (8)	0.086 (8)	−0.001 (7)	0.063 (7)	−0.005 (7)
O1AA	0.032 (3)	0.028 (2)	0.0386 (14)	−0.0019 (17)	0.012 (2)	−0.0017 (13)
C1AA	0.037 (2)	0.039 (2)	0.049 (2)	0.0004 (18)	0.0110 (15)	−0.0104 (16)
C2AA	0.063 (3)	0.064 (4)	0.075 (3)	0.004 (3)	0.044 (3)	0.001 (3)
O1AB	0.037 (5)	0.036 (4)	0.038 (3)	−0.001 (3)	0.003 (3)	−0.006 (3)
C1AB	0.057 (5)	0.073 (7)	0.080 (6)	−0.012 (5)	0.027 (4)	−0.040 (5)
C2AB	0.063 (5)	0.101 (11)	0.099 (7)	−0.021 (6)	0.043 (5)	−0.040 (7)

### Geometric parameters (Å, °)

**Table:**

O1A—C3A	1.4401 (19)	C4B—C5B	1.563 (2)
O1A—H11	0.85 (3)	C5B—C6B	1.531 (2)
O2A—C28A	1.213 (2)	C5B—C10B	1.557 (2)
O3A—C28A	1.319 (2)	C5B—H5B	0.9800
O3A—H31	0.95 (3)	C6B—C7B	1.532 (2)
C1A—C2A	1.533 (2)	C6B—H6C	0.9700
C1A—C10A	1.538 (2)	C6B—H6D	0.9700
C1A—H1A	0.9700	C7B—C8B	1.541 (2)
C1A—H1B	0.9700	C7B—H7C	0.9700
C2A—C3A	1.511 (2)	C7B—H7D	0.9700
C2A—H2A	0.9700	C8B—C26B	1.546 (2)
C2A—H2B	0.9700	C8B—C9B	1.558 (2)
C3A—C4A	1.535 (2)	C8B—C14B	1.594 (2)
C3A—H3A	0.9800	C9B—C11B	1.540 (2)
C4A—C23A	1.539 (2)	C9B—C10B	1.568 (2)
C4A—C24A	1.539 (2)	C9B—H9B	0.9800
C4A—C5A	1.5591 (19)	C10B—C25B	1.545 (2)
C5A—C6A	1.525 (2)	C11B—C12B	1.503 (2)
C5A—C10A	1.559 (2)	C11B—H11C	0.9700
C5A—H5A	0.9800	C11B—H11D	0.9700
C6A—C7A	1.527 (2)	C12B—C13B	1.331 (3)
C6A—H6A	0.9700	C12B—H12B	0.9300
C6A—H6B	0.9700	C13B—C18B	1.529 (2)
C7A—C8A	1.541 (2)	C13B—C14B	1.531 (2)
C7A—H7A	0.9700	C14B—C27B	1.549 (2)
C7A—H7B	0.9700	C14B—C15B	1.553 (2)
C8A—C26A	1.547 (2)	C15B—C16B	1.533 (2)
C8A—C9A	1.555 (2)	C15B—H15C	0.9700
C8A—C14A	1.588 (2)	C15B—H15D	0.9700
C9A—C11A	1.542 (2)	C16B—C17B	1.523 (3)
C9A—C10A	1.573 (2)	C16B—H16C	0.9700
C9A—H9A	0.9800	C16B—H16D	0.9700
C10A—C25A	1.539 (2)	C17B—C28B	1.532 (2)
C11A—C12A	1.509 (2)	C17B—C18B	1.545 (2)
C11A—H11A	0.9700	C17B—C22B	1.557 (2)
C11A—H11B	0.9700	C18B—C19B	1.545 (2)
C12A—C13A	1.324 (2)	C18B—H18B	0.9800
C12A—H12A	0.9300	C19B—C20B	1.541 (2)
C13A—C18A	1.524 (2)	C19B—H19C	0.9700
C13A—C14A	1.537 (2)	C19B—H19D	0.9700
C14A—C15A	1.544 (2)	C20B—C30B	1.526 (3)
C14A—C27A	1.560 (2)	C20B—C29B	1.527 (3)
C15A—C16A	1.524 (2)	C20B—C21B	1.538 (3)
C15A—H15A	0.9700	C21B—C22B	1.528 (3)
C15A—H15B	0.9700	C21B—H21C	0.9700
C16A—C17A	1.534 (2)	C21B—H21D	0.9700
C16A—H16A	0.9700	C22B—H22C	0.9700
C16A—H16B	0.9700	C22B—H22D	0.9700
C17A—C28A	1.525 (2)	C23B—H23D	0.9600
C17A—C18A	1.545 (2)	C23B—H23E	0.9600
C17A—C22A	1.557 (2)	C23B—H23F	0.9600
C18A—C19A	1.545 (2)	C24B—H24D	0.9600
C18A—H18A	0.9800	C24B—H24E	0.9600
C19A—C20A	1.538 (2)	C24B—H24F	0.9600
C19A—H19A	0.9700	C25B—H25D	0.9600
C19A—H19B	0.9700	C25B—H25E	0.9600
C20A—C21A	1.529 (2)	C25B—H25F	0.9600
C20A—C30A	1.531 (2)	C26B—H26D	0.9600
C20A—C29A	1.534 (2)	C26B—H26E	0.9600
C21A—C22A	1.522 (2)	C26B—H26F	0.9600
C21A—H21A	0.9700	C27B—H27D	0.9600
C21A—H21B	0.9700	C27B—H27E	0.9600
C22A—H22A	0.9700	C27B—H27F	0.9600
C22A—H22B	0.9700	C29B—H29D	0.9600
C23A—H23A	0.9600	C29B—H29E	0.9600
C23A—H23B	0.9600	C29B—H29F	0.9600
C23A—H23C	0.9600	C30B—H30D	0.9600
C24A—H24A	0.9600	C30B—H30E	0.9600
C24A—H24B	0.9600	C30B—H30F	0.9600
C24A—H24C	0.9600	O1BA—C1BA	1.418 (4)
C25A—H25A	0.9600	O1BA—C1BB	1.451 (10)
C25A—H25B	0.9600	O1BA—H1BO	0.84 (3)
C25A—H25C	0.9600	C1BA—C2BA	1.487 (6)
C26A—H26A	0.9600	C1BA—H1BA	0.9700
C26A—H26B	0.9600	C1BA—H1BB	0.9700
C26A—H26C	0.9600	C2BA—H2BA	0.9600
C27A—H27A	0.9600	C2BA—H2BB	0.9600
C27A—H27B	0.9600	C2BA—H2BC	0.9600
C27A—H27C	0.9600	C1BB—C2BB	1.522 (15)
C29A—H29A	0.9600	C1BB—H1BC	0.9700
C29A—H29B	0.9600	C1BB—H1BD	0.9700
C29A—H29C	0.9600	C2BB—H2BD	0.9600
C30A—H30A	0.9600	C2BB—H2BE	0.9600
C30A—H30B	0.9600	C2BB—H2BF	0.9600
C30A—H30C	0.9600	O1AA—C1AA	1.422 (7)
O1B—C3B	1.4373 (19)	O1AA—H1AO	0.8200
O1B—H12	0.83 (3)	C1AA—C2AA	1.487 (7)
O2B—C28B	1.218 (2)	C1AA—H1AA	0.9700
O3B—C28B	1.317 (3)	C1AA—H1AB	0.9700
O3B—H32	0.96 (4)	C2AA—H2AA	0.9600
C1B—C2B	1.530 (2)	C2AA—H2AB	0.9600
C1B—C10B	1.538 (2)	C2AA—H2AC	0.9600
C1B—H1C	0.9700	O1AB—C1AB	1.466 (13)
C1B—H1D	0.9700	O1AB—H2AO	0.8200
C2B—C3B	1.509 (2)	C1AB—C2AB	1.467 (15)
C2B—H2C	0.9700	C1AB—H1AC	0.9700
C2B—H2D	0.9700	C1AB—H1AD	0.9700
C3B—C4B	1.551 (2)	C2AB—H2AD	0.9600
C3B—H3B	0.9800	C2AB—H2AE	0.9600
C4B—C24B	1.531 (2)	C2AB—H2AF	0.9600
C4B—C23B	1.534 (2)		
			
C3A—O1A—H11	111 (2)	C6B—C5B—C10B	109.91 (13)
C28A—O3A—H31	110 (2)	C6B—C5B—C4B	114.05 (13)
C2A—C1A—C10A	113.64 (13)	C10B—C5B—C4B	117.90 (13)
C2A—C1A—H1A	108.8	C6B—C5B—H5B	104.5
C10A—C1A—H1A	108.8	C10B—C5B—H5B	104.5
C2A—C1A—H1B	108.8	C4B—C5B—H5B	104.5
C10A—C1A—H1B	108.8	C5B—C6B—C7B	110.55 (13)
H1A—C1A—H1B	107.7	C5B—C6B—H6C	109.5
C3A—C2A—C1A	110.93 (13)	C7B—C6B—H6C	109.5
C3A—C2A—H2A	109.5	C5B—C6B—H6D	109.5
C1A—C2A—H2A	109.5	C7B—C6B—H6D	109.5
C3A—C2A—H2B	109.5	H6C—C6B—H6D	108.1
C1A—C2A—H2B	109.5	C6B—C7B—C8B	114.57 (14)
H2A—C2A—H2B	108.0	C6B—C7B—H7C	108.6
O1A—C3A—C2A	106.81 (13)	C8B—C7B—H7C	108.6
O1A—C3A—C4A	112.41 (14)	C6B—C7B—H7D	108.6
C2A—C3A—C4A	113.71 (13)	C8B—C7B—H7D	108.6
O1A—C3A—H3A	107.9	H7C—C7B—H7D	107.6
C2A—C3A—H3A	107.9	C7B—C8B—C26B	108.41 (14)
C4A—C3A—H3A	107.9	C7B—C8B—C9B	109.85 (13)
C3A—C4A—C23A	108.20 (13)	C26B—C8B—C9B	110.60 (14)
C3A—C4A—C24A	111.30 (14)	C7B—C8B—C14B	110.08 (14)
C23A—C4A—C24A	107.86 (14)	C26B—C8B—C14B	110.05 (13)
C3A—C4A—C5A	106.67 (13)	C9B—C8B—C14B	107.85 (13)
C23A—C4A—C5A	107.83 (13)	C11B—C9B—C8B	110.07 (13)
C24A—C4A—C5A	114.78 (13)	C11B—C9B—C10B	113.45 (13)
C6A—C5A—C10A	109.99 (12)	C8B—C9B—C10B	116.98 (13)
C6A—C5A—C4A	114.28 (13)	C11B—C9B—H9B	105.0
C10A—C5A—C4A	117.30 (13)	C8B—C9B—H9B	105.0
C6A—C5A—H5A	104.6	C10B—C9B—H9B	105.0
C10A—C5A—H5A	104.6	C1B—C10B—C25B	107.16 (13)
C4A—C5A—H5A	104.6	C1B—C10B—C5B	108.24 (12)
C5A—C6A—C7A	110.16 (13)	C25B—C10B—C5B	113.20 (13)
C5A—C6A—H6A	109.6	C1B—C10B—C9B	107.99 (12)
C7A—C6A—H6A	109.6	C25B—C10B—C9B	113.89 (13)
C5A—C6A—H6B	109.6	C5B—C10B—C9B	106.15 (13)
C7A—C6A—H6B	109.6	C12B—C11B—C9B	114.41 (14)
H6A—C6A—H6B	108.1	C12B—C11B—H11C	108.7
C6A—C7A—C8A	113.76 (13)	C9B—C11B—H11C	108.7
C6A—C7A—H7A	108.8	C12B—C11B—H11D	108.7
C8A—C7A—H7A	108.8	C9B—C11B—H11D	108.7
C6A—C7A—H7B	108.8	H11C—C11B—H11D	107.6
C8A—C7A—H7B	108.8	C13B—C12B—C11B	125.63 (16)
H7A—C7A—H7B	107.7	C13B—C12B—H12B	117.2
C7A—C8A—C26A	107.68 (13)	C11B—C12B—H12B	117.2
C7A—C8A—C9A	110.93 (12)	C12B—C13B—C18B	118.76 (16)
C26A—C8A—C9A	111.13 (13)	C12B—C13B—C14B	120.79 (15)
C7A—C8A—C14A	109.49 (12)	C18B—C13B—C14B	120.37 (15)
C26A—C8A—C14A	110.41 (12)	C13B—C14B—C27B	106.83 (13)
C9A—C8A—C14A	107.20 (13)	C13B—C14B—C15B	112.09 (13)
C11A—C9A—C8A	109.91 (12)	C27B—C14B—C15B	107.32 (14)
C11A—C9A—C10A	113.88 (13)	C13B—C14B—C8B	108.83 (14)
C8A—C9A—C10A	117.33 (12)	C27B—C14B—C8B	112.92 (13)
C11A—C9A—H9A	104.8	C15B—C14B—C8B	108.89 (13)
C8A—C9A—H9A	104.8	C16B—C15B—C14B	115.71 (14)
C10A—C9A—H9A	104.8	C16B—C15B—H15C	108.4
C1A—C10A—C25A	108.01 (13)	C14B—C15B—H15C	108.4
C1A—C10A—C5A	108.34 (12)	C16B—C15B—H15D	108.4
C25A—C10A—C5A	113.54 (13)	C14B—C15B—H15D	108.4
C1A—C10A—C9A	107.50 (12)	H15C—C15B—H15D	107.4
C25A—C10A—C9A	113.78 (12)	C17B—C16B—C15B	112.61 (15)
C5A—C10A—C9A	105.41 (12)	C17B—C16B—H16C	109.1
C12A—C11A—C9A	113.60 (13)	C15B—C16B—H16C	109.1
C12A—C11A—H11A	108.8	C17B—C16B—H16D	109.1
C9A—C11A—H11A	108.8	C15B—C16B—H16D	109.1
C12A—C11A—H11B	108.8	H16C—C16B—H16D	107.8
C9A—C11A—H11B	108.8	C16B—C17B—C28B	110.88 (14)
H11A—C11A—H11B	107.7	C16B—C17B—C18B	109.61 (14)
C13A—C12A—C11A	126.24 (15)	C28B—C17B—C18B	110.91 (15)
C13A—C12A—H12A	116.9	C16B—C17B—C22B	111.36 (15)
C11A—C12A—H12A	116.9	C28B—C17B—C22B	103.61 (13)
C12A—C13A—C18A	119.28 (14)	C18B—C17B—C22B	110.35 (14)
C12A—C13A—C14A	120.18 (14)	C13B—C18B—C19B	113.21 (13)
C18A—C13A—C14A	120.44 (13)	C13B—C18B—C17B	110.87 (14)
C13A—C14A—C15A	112.98 (12)	C19B—C18B—C17B	111.48 (14)
C13A—C14A—C27A	106.66 (13)	C13B—C18B—H18B	107.0
C15A—C14A—C27A	106.56 (13)	C19B—C18B—H18B	107.0
C13A—C14A—C8A	107.86 (12)	C17B—C18B—H18B	107.0
C15A—C14A—C8A	109.88 (13)	C20B—C19B—C18B	114.44 (14)
C27A—C14A—C8A	112.96 (12)	C20B—C19B—H19C	108.7
C16A—C15A—C14A	114.39 (13)	C18B—C19B—H19C	108.7
C16A—C15A—H15A	108.7	C20B—C19B—H19D	108.7
C14A—C15A—H15A	108.7	C18B—C19B—H19D	108.7
C16A—C15A—H15B	108.7	H19C—C19B—H19D	107.6
C14A—C15A—H15B	108.7	C30B—C20B—C29B	109.03 (16)
H15A—C15A—H15B	107.6	C30B—C20B—C21B	110.73 (16)
C15A—C16A—C17A	111.58 (13)	C29B—C20B—C21B	108.66 (17)
C15A—C16A—H16A	109.3	C30B—C20B—C19B	111.38 (17)
C17A—C16A—H16A	109.3	C29B—C20B—C19B	108.71 (15)
C15A—C16A—H16B	109.3	C21B—C20B—C19B	108.27 (15)
C17A—C16A—H16B	109.3	C22B—C21B—C20B	112.96 (17)
H16A—C16A—H16B	108.0	C22B—C21B—H21C	109.0
C28A—C17A—C16A	111.69 (13)	C20B—C21B—H21C	109.0
C28A—C17A—C18A	109.38 (13)	C22B—C21B—H21D	109.0
C16A—C17A—C18A	109.48 (12)	C20B—C21B—H21D	109.0
C28A—C17A—C22A	104.57 (12)	H21C—C21B—H21D	107.8
C16A—C17A—C22A	110.46 (13)	C21B—C22B—C17B	112.89 (15)
C18A—C17A—C22A	111.20 (13)	C21B—C22B—H22C	109.0
C13A—C18A—C19A	111.57 (12)	C17B—C22B—H22C	109.0
C13A—C18A—C17A	112.13 (12)	C21B—C22B—H22D	109.0
C19A—C18A—C17A	112.01 (13)	C17B—C22B—H22D	109.0
C13A—C18A—H18A	106.9	H22C—C22B—H22D	107.8
C19A—C18A—H18A	106.9	C4B—C23B—H23D	109.5
C17A—C18A—H18A	106.9	C4B—C23B—H23E	109.5
C20A—C19A—C18A	113.95 (13)	H23D—C23B—H23E	109.5
C20A—C19A—H19A	108.8	C4B—C23B—H23F	109.5
C18A—C19A—H19A	108.8	H23D—C23B—H23F	109.5
C20A—C19A—H19B	108.8	H23E—C23B—H23F	109.5
C18A—C19A—H19B	108.8	C4B—C24B—H24D	109.5
H19A—C19A—H19B	107.7	C4B—C24B—H24E	109.5
C21A—C20A—C30A	110.37 (14)	H24D—C24B—H24E	109.5
C21A—C20A—C29A	108.66 (14)	C4B—C24B—H24F	109.5
C30A—C20A—C29A	108.63 (15)	H24D—C24B—H24F	109.5
C21A—C20A—C19A	108.14 (13)	H24E—C24B—H24F	109.5
C30A—C20A—C19A	111.38 (13)	C10B—C25B—H25D	109.5
C29A—C20A—C19A	109.62 (13)	C10B—C25B—H25E	109.5
C22A—C21A—C20A	112.43 (13)	H25D—C25B—H25E	109.5
C22A—C21A—H21A	109.1	C10B—C25B—H25F	109.5
C20A—C21A—H21A	109.1	H25D—C25B—H25F	109.5
C22A—C21A—H21B	109.1	H25E—C25B—H25F	109.5
C20A—C21A—H21B	109.1	C8B—C26B—H26D	109.5
H21A—C21A—H21B	107.8	C8B—C26B—H26E	109.5
C21A—C22A—C17A	112.69 (13)	H26D—C26B—H26E	109.5
C21A—C22A—H22A	109.1	C8B—C26B—H26F	109.5
C17A—C22A—H22A	109.1	H26D—C26B—H26F	109.5
C21A—C22A—H22B	109.1	H26E—C26B—H26F	109.5
C17A—C22A—H22B	109.1	C14B—C27B—H27D	109.5
H22A—C22A—H22B	107.8	C14B—C27B—H27E	109.5
C4A—C23A—H23A	109.5	H27D—C27B—H27E	109.5
C4A—C23A—H23B	109.5	C14B—C27B—H27F	109.5
H23A—C23A—H23B	109.5	H27D—C27B—H27F	109.5
C4A—C23A—H23C	109.5	H27E—C27B—H27F	109.5
H23A—C23A—H23C	109.5	O2B—C28B—O3B	122.88 (18)
H23B—C23A—H23C	109.5	O2B—C28B—C17B	123.32 (18)
C4A—C24A—H24A	109.5	O3B—C28B—C17B	113.62 (16)
C4A—C24A—H24B	109.5	C20B—C29B—H29D	109.5
H24A—C24A—H24B	109.5	C20B—C29B—H29E	109.5
C4A—C24A—H24C	109.5	H29D—C29B—H29E	109.5
H24A—C24A—H24C	109.5	C20B—C29B—H29F	109.5
H24B—C24A—H24C	109.5	H29D—C29B—H29F	109.5
C10A—C25A—H25A	109.5	H29E—C29B—H29F	109.5
C10A—C25A—H25B	109.5	C20B—C30B—H30D	109.5
H25A—C25A—H25B	109.5	C20B—C30B—H30E	109.5
C10A—C25A—H25C	109.5	H30D—C30B—H30E	109.5
H25A—C25A—H25C	109.5	C20B—C30B—H30F	109.5
H25B—C25A—H25C	109.5	H30D—C30B—H30F	109.5
C8A—C26A—H26A	109.5	H30E—C30B—H30F	109.5
C8A—C26A—H26B	109.5	C1BA—O1BA—C1BB	30.4 (5)
H26A—C26A—H26B	109.5	C1BA—O1BA—H1BO	114 (2)
C8A—C26A—H26C	109.5	C1BB—O1BA—H1BO	107 (2)
H26A—C26A—H26C	109.5	O1BA—C1BA—C2BA	111.7 (4)
H26B—C26A—H26C	109.5	O1BA—C1BA—H1BA	109.3
C14A—C27A—H27A	109.5	C2BA—C1BA—H1BA	109.3
C14A—C27A—H27B	109.5	O1BA—C1BA—H1BB	109.3
H27A—C27A—H27B	109.5	C2BA—C1BA—H1BB	109.3
C14A—C27A—H27C	109.5	H1BA—C1BA—H1BB	107.9
H27A—C27A—H27C	109.5	C1BA—C2BA—H2BA	109.5
H27B—C27A—H27C	109.5	C1BA—C2BA—H2BB	109.5
O2A—C28A—O3A	121.74 (16)	H2BA—C2BA—H2BB	109.5
O2A—C28A—C17A	124.65 (15)	C1BA—C2BA—H2BC	109.5
O3A—C28A—C17A	113.51 (14)	H2BA—C2BA—H2BC	109.5
C20A—C29A—H29A	109.5	H2BB—C2BA—H2BC	109.5
C20A—C29A—H29B	109.5	O1BA—C1BB—C2BB	106.6 (11)
H29A—C29A—H29B	109.5	O1BA—C1BB—H1BC	110.4
C20A—C29A—H29C	109.5	C2BB—C1BB—H1BC	110.4
H29A—C29A—H29C	109.5	O1BA—C1BB—H1BD	110.4
H29B—C29A—H29C	109.5	C2BB—C1BB—H1BD	110.4
C20A—C30A—H30A	109.5	H1BC—C1BB—H1BD	108.6
C20A—C30A—H30B	109.5	C1BB—C2BB—H2BD	109.5
H30A—C30A—H30B	109.5	C1BB—C2BB—H2BE	109.5
C20A—C30A—H30C	109.5	H2BD—C2BB—H2BE	109.5
H30A—C30A—H30C	109.5	C1BB—C2BB—H2BF	109.5
H30B—C30A—H30C	109.5	H2BD—C2BB—H2BF	109.5
C3B—O1B—H12	112.0 (17)	H2BE—C2BB—H2BF	109.5
C28B—O3B—H32	107 (3)	O1AA—C1AA—C2AA	111.4 (4)
C2B—C1B—C10B	113.09 (13)	O1AA—C1AA—H1AA	109.3
C2B—C1B—H1C	109.0	C2AA—C1AA—H1AA	109.3
C10B—C1B—H1C	109.0	O1AA—C1AA—H1AB	109.3
C2B—C1B—H1D	109.0	C2AA—C1AA—H1AB	109.3
C10B—C1B—H1D	109.0	H1AA—C1AA—H1AB	108.0
H1C—C1B—H1D	107.8	C1AA—C2AA—H2AA	109.5
C3B—C2B—C1B	111.29 (13)	C1AA—C2AA—H2AB	109.5
C3B—C2B—H2C	109.4	H2AA—C2AA—H2AB	109.5
C1B—C2B—H2C	109.4	C1AA—C2AA—H2AC	109.5
C3B—C2B—H2D	109.4	H2AA—C2AA—H2AC	109.5
C1B—C2B—H2D	109.4	H2AB—C2AA—H2AC	109.5
H2C—C2B—H2D	108.0	C1AB—O1AB—H2AO	109.5
O1B—C3B—C2B	107.66 (13)	O1AB—C1AB—C2AB	109.2 (9)
O1B—C3B—C4B	111.10 (13)	O1AB—C1AB—H1AC	109.9
C2B—C3B—C4B	113.90 (13)	C2AB—C1AB—H1AC	109.9
O1B—C3B—H3B	108.0	O1AB—C1AB—H1AD	109.9
C2B—C3B—H3B	108.0	C2AB—C1AB—H1AD	109.9
C4B—C3B—H3B	108.0	H1AC—C1AB—H1AD	108.3
C24B—C4B—C23B	107.98 (15)	C1AB—C2AB—H2AD	109.5
C24B—C4B—C3B	111.04 (14)	C1AB—C2AB—H2AE	109.5
C23B—C4B—C3B	107.20 (13)	H2AD—C2AB—H2AE	109.5
C24B—C4B—C5B	114.26 (13)	C1AB—C2AB—H2AF	109.5
C23B—C4B—C5B	108.69 (13)	H2AD—C2AB—H2AF	109.5
C3B—C4B—C5B	107.43 (13)	H2AE—C2AB—H2AF	109.5
			
C10A—C1A—C2A—C3A	−55.77 (18)	C1B—C2B—C3B—O1B	−178.54 (12)
C1A—C2A—C3A—O1A	−176.66 (13)	C1B—C2B—C3B—C4B	57.79 (18)
C1A—C2A—C3A—C4A	58.75 (18)	O1B—C3B—C4B—C24B	−47.93 (18)
O1A—C3A—C4A—C23A	67.63 (17)	C2B—C3B—C4B—C24B	73.85 (17)
C2A—C3A—C4A—C23A	−170.86 (13)	O1B—C3B—C4B—C23B	69.80 (17)
O1A—C3A—C4A—C24A	−50.70 (17)	C2B—C3B—C4B—C23B	−168.41 (14)
C2A—C3A—C4A—C24A	70.81 (17)	O1B—C3B—C4B—C5B	−173.53 (13)
O1A—C3A—C4A—C5A	−176.59 (12)	C2B—C3B—C4B—C5B	−51.75 (17)
C2A—C3A—C4A—C5A	−55.08 (17)	C24B—C4B—C5B—C6B	57.20 (19)
C3A—C4A—C5A—C6A	−176.53 (13)	C23B—C4B—C5B—C6B	−63.45 (18)
C23A—C4A—C5A—C6A	−60.50 (17)	C3B—C4B—C5B—C6B	−179.14 (14)
C24A—C4A—C5A—C6A	59.71 (19)	C24B—C4B—C5B—C10B	−73.97 (18)
C3A—C4A—C5A—C10A	52.57 (17)	C23B—C4B—C5B—C10B	165.37 (13)
C23A—C4A—C5A—C10A	168.59 (14)	C3B—C4B—C5B—C10B	49.69 (17)
C24A—C4A—C5A—C10A	−71.19 (19)	C10B—C5B—C6B—C7B	−63.43 (17)
C10A—C5A—C6A—C7A	−65.99 (16)	C4B—C5B—C6B—C7B	161.60 (14)
C4A—C5A—C6A—C7A	159.62 (12)	C5B—C6B—C7B—C8B	55.46 (19)
C5A—C6A—C7A—C8A	56.35 (17)	C6B—C7B—C8B—C26B	75.53 (17)
C6A—C7A—C8A—C26A	77.31 (16)	C6B—C7B—C8B—C9B	−45.43 (18)
C6A—C7A—C8A—C9A	−44.50 (18)	C6B—C7B—C8B—C14B	−164.04 (13)
C6A—C7A—C8A—C14A	−162.62 (13)	C7B—C8B—C9B—C11B	178.20 (14)
C7A—C8A—C9A—C11A	176.98 (13)	C26B—C8B—C9B—C11B	58.57 (17)
C26A—C8A—C9A—C11A	57.21 (16)	C14B—C8B—C9B—C11B	−61.82 (17)
C14A—C8A—C9A—C11A	−63.52 (15)	C7B—C8B—C9B—C10B	46.79 (18)
C7A—C8A—C9A—C10A	44.78 (17)	C26B—C8B—C9B—C10B	−72.84 (17)
C26A—C8A—C9A—C10A	−74.99 (16)	C14B—C8B—C9B—C10B	166.77 (13)
C14A—C8A—C9A—C10A	164.28 (12)	C2B—C1B—C10B—C25B	−70.40 (17)
C2A—C1A—C10A—C25A	−73.17 (16)	C2B—C1B—C10B—C5B	52.00 (17)
C2A—C1A—C10A—C5A	50.21 (17)	C2B—C1B—C10B—C9B	166.52 (13)
C2A—C1A—C10A—C9A	163.67 (13)	C6B—C5B—C10B—C1B	176.57 (13)
C6A—C5A—C10A—C1A	176.33 (12)	C4B—C5B—C10B—C1B	−50.41 (17)
C4A—C5A—C10A—C1A	−50.82 (17)	C6B—C5B—C10B—C25B	−64.80 (17)
C6A—C5A—C10A—C25A	−63.69 (16)	C4B—C5B—C10B—C25B	68.22 (17)
C4A—C5A—C10A—C25A	69.16 (17)	C6B—C5B—C10B—C9B	60.85 (15)
C6A—C5A—C10A—C9A	61.51 (15)	C4B—C5B—C10B—C9B	−166.13 (12)
C4A—C5A—C10A—C9A	−165.64 (13)	C11B—C9B—C10B—C1B	59.73 (17)
C11A—C9A—C10A—C1A	61.57 (17)	C8B—C9B—C10B—C1B	−170.44 (13)
C8A—C9A—C10A—C1A	−168.05 (12)	C11B—C9B—C10B—C25B	−59.15 (18)
C11A—C9A—C10A—C25A	−57.98 (18)	C8B—C9B—C10B—C25B	70.68 (18)
C8A—C9A—C10A—C25A	72.40 (17)	C11B—C9B—C10B—C5B	175.62 (13)
C11A—C9A—C10A—C5A	176.98 (13)	C8B—C9B—C10B—C5B	−54.54 (16)
C8A—C9A—C10A—C5A	−52.64 (16)	C8B—C9B—C11B—C12B	35.28 (19)
C8A—C9A—C11A—C12A	34.48 (19)	C10B—C9B—C11B—C12B	168.52 (13)
C10A—C9A—C11A—C12A	168.45 (13)	C9B—C11B—C12B—C13B	−5.0 (3)
C9A—C11A—C12A—C13A	−3.4 (2)	C11B—C12B—C13B—C18B	179.16 (15)
C11A—C12A—C13A—C18A	178.90 (15)	C11B—C12B—C13B—C14B	2.5 (3)
C11A—C12A—C13A—C14A	2.5 (3)	C12B—C13B—C14B—C27B	92.89 (19)
C12A—C13A—C14A—C15A	−153.16 (15)	C18B—C13B—C14B—C27B	−83.77 (17)
C18A—C13A—C14A—C15A	30.47 (19)	C12B—C13B—C14B—C15B	−149.83 (16)
C12A—C13A—C14A—C27A	90.09 (17)	C18B—C13B—C14B—C15B	33.5 (2)
C18A—C13A—C14A—C27A	−86.28 (16)	C12B—C13B—C14B—C8B	−29.3 (2)
C12A—C13A—C14A—C8A	−31.5 (2)	C18B—C13B—C14B—C8B	154.02 (13)
C18A—C13A—C14A—C8A	152.11 (13)	C7B—C8B—C14B—C13B	177.90 (13)
C7A—C8A—C14A—C13A	−178.58 (12)	C26B—C8B—C14B—C13B	−62.66 (17)
C26A—C8A—C14A—C13A	−60.20 (17)	C9B—C8B—C14B—C13B	58.07 (16)
C9A—C8A—C14A—C13A	61.00 (15)	C7B—C8B—C14B—C27B	59.45 (18)
C7A—C8A—C14A—C15A	−55.04 (16)	C26B—C8B—C14B—C27B	178.88 (15)
C26A—C8A—C14A—C15A	63.35 (16)	C9B—C8B—C14B—C27B	−60.38 (18)
C9A—C8A—C14A—C15A	−175.46 (12)	C7B—C8B—C14B—C15B	−59.64 (17)
C7A—C8A—C14A—C27A	63.80 (17)	C26B—C8B—C14B—C15B	59.79 (18)
C26A—C8A—C14A—C27A	−177.82 (14)	C9B—C8B—C14B—C15B	−179.47 (13)
C9A—C8A—C14A—C27A	−56.62 (16)	C13B—C14B—C15B—C16B	−35.3 (2)
C13A—C14A—C15A—C16A	−37.70 (18)	C27B—C14B—C15B—C16B	81.70 (18)
C27A—C14A—C15A—C16A	79.11 (16)	C8B—C14B—C15B—C16B	−155.77 (14)
C8A—C14A—C15A—C16A	−158.19 (12)	C14B—C15B—C16B—C17B	50.7 (2)
C14A—C15A—C16A—C17A	55.35 (18)	C15B—C16B—C17B—C28B	62.82 (18)
C15A—C16A—C17A—C28A	59.27 (18)	C15B—C16B—C17B—C18B	−59.97 (18)
C15A—C16A—C17A—C18A	−62.03 (17)	C15B—C16B—C17B—C22B	177.62 (13)
C15A—C16A—C17A—C22A	175.20 (13)	C12B—C13B—C18B—C19B	−94.9 (2)
C12A—C13A—C18A—C19A	−88.40 (18)	C14B—C13B—C18B—C19B	81.82 (19)
C14A—C13A—C18A—C19A	88.01 (17)	C12B—C13B—C18B—C17B	138.96 (17)
C12A—C13A—C18A—C17A	145.03 (15)	C14B—C13B—C18B—C17B	−44.3 (2)
C14A—C13A—C18A—C17A	−38.57 (19)	C16B—C17B—C18B—C13B	55.20 (18)
C28A—C17A—C18A—C13A	−70.36 (15)	C28B—C17B—C18B—C13B	−67.57 (19)
C16A—C17A—C18A—C13A	52.32 (16)	C22B—C17B—C18B—C13B	178.20 (15)
C22A—C17A—C18A—C13A	174.65 (12)	C16B—C17B—C18B—C19B	−71.90 (18)
C28A—C17A—C18A—C19A	163.31 (12)	C28B—C17B—C18B—C19B	165.33 (15)
C16A—C17A—C18A—C19A	−74.01 (15)	C22B—C17B—C18B—C19B	51.1 (2)
C22A—C17A—C18A—C19A	48.32 (16)	C13B—C18B—C19B—C20B	179.45 (16)
C13A—C18A—C19A—C20A	−179.70 (13)	C17B—C18B—C19B—C20B	−54.7 (2)
C17A—C18A—C19A—C20A	−53.06 (17)	C18B—C19B—C20B—C30B	−67.1 (2)
C18A—C19A—C20A—C21A	56.21 (17)	C18B—C19B—C20B—C29B	172.74 (17)
C18A—C19A—C20A—C30A	−65.23 (19)	C18B—C19B—C20B—C21B	54.9 (2)
C18A—C19A—C20A—C29A	174.52 (15)	C30B—C20B—C21B—C22B	67.6 (2)
C30A—C20A—C21A—C22A	64.42 (17)	C29B—C20B—C21B—C22B	−172.67 (17)
C29A—C20A—C21A—C22A	−176.56 (14)	C19B—C20B—C21B—C22B	−54.8 (2)
C19A—C20A—C21A—C22A	−57.64 (17)	C20B—C21B—C22B—C17B	55.9 (2)
C20A—C21A—C22A—C17A	56.85 (18)	C16B—C17B—C22B—C21B	69.4 (2)
C28A—C17A—C22A—C21A	−168.81 (14)	C28B—C17B—C22B—C21B	−171.41 (17)
C16A—C17A—C22A—C21A	70.90 (17)	C18B—C17B—C22B—C21B	−52.6 (2)
C18A—C17A—C22A—C21A	−50.87 (18)	C16B—C17B—C28B—O2B	23.8 (2)
C16A—C17A—C28A—O2A	−148.96 (15)	C18B—C17B—C28B—O2B	145.84 (16)
C18A—C17A—C28A—O2A	−27.6 (2)	C22B—C17B—C28B—O2B	−95.76 (19)
C22A—C17A—C28A—O2A	91.56 (18)	C16B—C17B—C28B—O3B	−160.88 (15)
C16A—C17A—C28A—O3A	34.65 (19)	C18B—C17B—C28B—O3B	−38.9 (2)
C18A—C17A—C28A—O3A	156.01 (13)	C22B—C17B—C28B—O3B	79.55 (19)
C22A—C17A—C28A—O3A	−84.82 (16)	C1BB—O1BA—C1BA—C2BA	−17.4 (10)
C10B—C1B—C2B—C3B	−57.70 (18)	C1BA—O1BA—C1BB—C2BB	−4.0 (7)

### Hydrogen-bond geometry (Å, °)

**Table:**

*D*—H···*A*	*D*—H	H···*A*	*D*···*A*	*D*—H···*A*
O1BA—H1BO···O1B	0.84 (3)	1.81 (3)	2.652 (2)	177 (3)
O1AA—H1AO···O1A	0.82	1.98	2.794 (7)	170
O1A—H11···O2B	0.85 (3)	2.02 (3)	2.8503 (18)	168 (3)
O1B—H12···O2A^i^	0.83 (3)	1.89 (3)	2.7204 (18)	174 (3)
O3A—H31···O1BA^ii^	0.95 (3)	1.61 (3)	2.552 (2)	177 (3)
O3B—H32···O1AA	0.96 (4)	1.64 (4)	2.575 (7)	163 (4)
C15A—H15A···O3A	0.97	2.58	3.1222 (19)	116
C15B—H15C···O2B	0.97	2.60	3.152 (2)	116
C23A—H23A···O2B	0.96	2.54	3.375 (2)	145

Symmetry codes: (i) *x*, *y*, *z*−1; (ii) *x*, *y*+1, *z*+1.
